# WU Polyomavirus in Children, Canada

**DOI:** 10.3201/eid1312.070909

**Published:** 2007-12

**Authors:** Yacine Abed, David Wang, Guy Boivin

**Affiliations:** * Centre Hospitalier Universitaire de Québec and Research Center in Infectious Diseases, Quebec City, Quebec, Canada; † Washington University School of Medicine, St. Louis, Missouri, USA

**Keywords:** WU polyomavirus, respiratory infections, children, PCR, dispatch

## Abstract

WU polyomavirus was detected in nasopharyngeal aspirates in 2 (2.5%) of 79 children with respiratory infections (both infected with respiratory syncytial virus) and in 5 (6.4%) of 78 asymptomatic children during the same winter season in Canada. The strains were closely related to Australian and American viruses based on analysis of large T antigen (TAg) and VP2 genes. The pathogenic role of WU virus is still uncertain.

Polyomaviruses are nonenveloped viruses that have an icosahedral capsid containing a small, circular, double-stranded DNA genome (*1*). These viruses have been identified in a variety of mammals and birds worldwide, and the most studied polyomavirus species infecting animals are the mouse polyomavirus (*2*) and the simian vacuolating (SV40) virus (*3*). In 1971, 2 human polyomavirus species named BK and JC viruses, respectively, were first isolated from the urine of a kidney allograft recipient with chronic pyelonephritis and advanced renal failure (*4*) and from the brain of a patient with progressive multifocal leukoencephalopathy (*5*). Recently, 2 new human polyomavirus members were described. The KI virus was identified in nasopharyngeal aspirates (NPA) and feces from patients with respiratory tract infections in Sweden (*6*). Also, Gaynor and colleagues (*7*) reported the detection and molecular characterization of the WU virus in clinical respiratory samples from patients with acute respiratory tract infections (ARTI). Although the pathogenesis of BK and JC viruses has been clearly established, the role of the KI and WU viruses as respiratory pathogens has yet to be demonstrated. In this article, we report on the molecular detection and characterization of WU viruses in NPA from hospitalized children with or without respiratory tract infections.

## The Study

We tested 157 NPA specimens obtained from a case-control study on the incidence of respiratory viral agents, the results of which have been partly reported by our group (*8*). Participants were children <3 years of age who were hospitalized from December 2002 through April 2003 at Laval University Hospital Center in Quebec City, Quebec, Canada. Case-patients were children admitted for ARTI (mostly bronchiolitis, pneumonitis, and laryngotracheobronchitis) who had an NPA collected as part of the investigation of their illness. A research nurse administered a specific questionnaire at admission in the presence of the parents. At the end of the hospitalization, the children’s charts were reviewed to collect clinical and laboratory data. Eligible controls were children hospitalized during the same period for any elective surgery. These children had no concomitant respiratory symptoms or fever at admission, although they might have had an ARTI in the weeks before hospitalization. The study nurse obtained a signed consent from parents and a NPA was obtained during surgery. The original study was approved by the ethics committee of the Center Hospitalier Universitaire de Quebec.

NPA samples (200 μL) were used for nucleic acid extraction using the QIAamp viral RNA Mini Kit (QIAGEN, Inc., Mississauga, Ontario, Canada), which has been shown to recover both RNA and DNA. These specimens were previously analyzed by using a multiplex real-time RT-PCR assay for influenza A and B viruses, human respiratory syncytial virus (hRSV), and human metapneumovirus (hMPV) (*8*). For symptomatic children, viral cultures and antigen detection assays were performed at the treating physician’s request. The specimens were frozen at –80°C during the 4 years before PCR studies began for WU polyomavirus. All specimens were first tested for WU virus DNA by using primers AG0048 and AG0049, which allowed the amplification of a 244-bp product in the 3′ end of the large T antigen (TAg) region (*7*). A plasmid containing the partial WU genomic DNA from the original Australian virus (B0 strain) served as the positive control in each PCR batch (*7*). For WU-positive samples with the TAg primers, a confirmatory PCR assay was performed with primers AG0044 and AG0045 to amplify a 250-bp fragment from the VP2 region (*7*). PCR products were analyzed by agarose gel electrophoresis. Positive amplicons were subsequently purified and sequenced by using the respective PCR primers.

The Canadian WU VP2 sequences were compared with those of 18 WU viruses that originated in Brisbane, Queensland, Australia, and St Louis, Missouri, USA (*7*). For this purpose, multiple nucleotide sequence alignments were performed by using the ClustalW program followed by phylogenetic analyses, which were conducted with the MEGA version 3.1 software using the neighbor-joining algorithm with Kimura-2 parameters (*9*).

By using the PCR assay with primers targeting the large TAg, WU sequences were detected in 2 (2.53%) of 79 symptomatic children (the 2 children were 13 months old) and in 5 (6.41%) of 78 asymptomatic children, 13–24 months of age (mean age 20 months) (Table). Symptomatic children had a diagnosis of bronchiolitis (patient 1) and pneumonitis (patient 2) and were both coinfected with hRSV. In contrast, no other viruses were detected in the asymptomatic children who underwent elective surgery. The duration of hospitalization for the 2 symptomatic children with dual WU/hRSV infection (3 and 4 days) was similar to that of 120 children with single hRSV infection (median: 4 days). By using the PCR assay with primers targeting the VP2 region, WU sequences were detected in 4/7 previously positive children, including 1 symptomatic (patient 1) and 3 asymptomatic (patients 3, 5 and 6) patients (Table). The 7 large TAg nt sequences of Canadian WU viruses were 100% identical and had 99.5% identity to the WU sequence contained in the control plasmid from Australia (data not shown). In addition, 100% identity was found between the VP2 nt sequences of the 4 Canadian WU viruses (data not shown). The latter also shared 100% identity with the most frequently observed WU genotypes (represented by previously-reported WU strains B9, S6, B28, B37, B22, B24, B35, B10, B1, and B17 [GenBank accession numbers: EF444592, EF444593, EF444590, EF444589, EF444588, EF444587, EF444586, EF444584, EF444583 and EF444582, respectively]) and, obviously, clustered together in the phylogenetic tree (Figure).

**Table:** Clinical data from 7 WU polyomavirus–infected children, Canada, 2003*

Patient no.	Sex	Age, mo	Date sample collected	Sample type	PCR for WU virus LTAg/VP2	Diagnosis	Copathogen
1	F	13	Feb 13	NPA	+/+	Bronchiolitis	hRSV
2	M	13	Mar 5	NPA	+/–	Pneumonitis	hRSV
3	F	13	Feb 24	NPA	+/+	None†	None
4	M	20	Mar 13	NPA	+/–	None†	None
5	F	24	Mar 17	NPA	+/+	None†	None
6	M	19	Mar 31	NPA	+/+	None†	None
7	F	24	Apr 1	NPA	+/–	None†	None

*NPA, nasopharyngeal aspirates; hRSV, human respiratory syncytial virus. †No fever or respiratory symptoms at the time of elective surgery.

**Figure:**
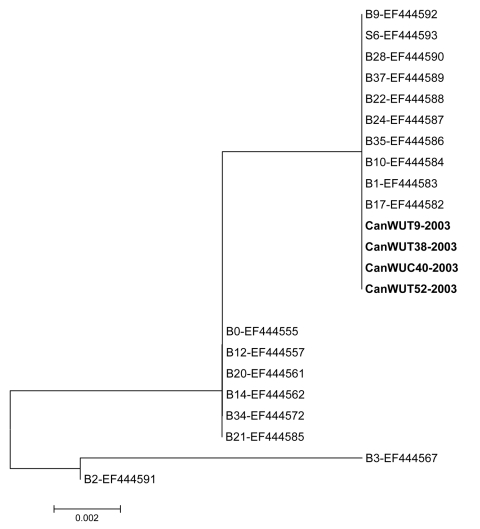
Phylogenetic analysis of Canadian WU polyomavirus strains CanWUT9–2003, CanWUT38–2003, CanWUC40–2003 and CanWUT52–2003 (shown in **boldface**), based on nucleotide sequences of the VP2 region. Multiple nucleotide sequence alignments were performed by using the ClustalW program and a phylogenetic tree was constructed with the MEGA 3.1 software using the neighbor-joining algorithm with Kimura-2 parameters (*9*). The analysis included WU strains previously identified from Australian and American cohorts (*7*) i.e., B9, S6, B28, B37, B22, B24, B35, B10, B1, B17, B0, B12, B20, B14, B34, B21, B3, and B2 (GenBank accession nos.: EF444592, EF444593, EF444590, EF444589, EF444588, EF444587, EF444586, EF444584, EF444583, EF444582, EF-444555, EF444557, EF444561, EF444562, EF444572, EF444585, EF444567, and EF444591, respectively).

## Conclusions

In this study, we report for the first time, to our knowledge, the presence of the newly described WU polyomavirus in Canadian children. We found that more asymptomatic (6.4%) than symptomatic (2.5%) children shed viral DNA in their respiratory tract. The WU polyomavirus was previously identified in respiratory tract samples from Australian and American patients, which suggests its worldwide distribution. Efforts to culture this new virus by using PCR-positive respiratory specimens have thus far been unsuccessful (D. Wang, unpub. data).

The 7 sequences of the large TAg region and the 4 sequences of the VP2 region from the Canadian WU strains displayed no sequence variations. This could be due to the short size of these PCR products (244 and 250 bp, respectively) and the stability of this double-stranded DNA genome. A similar finding of limited sequence variation was reported in the previous molecular study performed with Australian and American WU strains (*7*). Indeed, 4 Canadian WU strains had 100% nucleotide identity with 10 strains selected from these cohorts when VP2 sequences were compared (Figure).

Since we aimed at evaluating the possible contribution of the WU polyomavirus in respiratory tract infections of children, we tested NPA samples from symptomatic and asymptomatic subjects of the same age (<3 years) that were collected during the same winter period at the same institution. The WU virus was detected in 5 asymptomatic children at the time of an elective surgery and in 2 symptomatic children (bronchiolitis and pneumonitis) who were also infected with hRSV. A high rate of coinfection was also noted in the Australian cohort (68%) and in the American cohort (100%) (*7*). Notably, similar findings were obtained in a study on the related KI polyomavirus in which another viral pathogen was found in 5/6 KI-positive samples (*6*). By analogy with other human polyomaviruses (BK and JC), WU and KI possibly could establish a latent infection with subsequent asymptomatic reactivation; further studies are needed for confirmation. The presence of WU virus in the control children could also represent prolonged shedding from a prior respiratory tract infection. In conclusion, although this study confirmed the presence of the WU polyomavirus in NPA samples from Canadian children and suggests that its distribution is worldwide, its role in respiratory tract diseases of children remains undetermined.
